# Enhancing Podiatry Students' Understanding of Foot Arch Through Footprint Activity

**DOI:** 10.1002/jfa2.70151

**Published:** 2026-03-22

**Authors:** Angela Jacques, Fatemeh Chehrehasa

**Affiliations:** ^1^ Faculty of Health School of Biomedical Sciences Queensland University of Technology Brisbane Australia

**Keywords:** anatomical variation, foot arch, footprint analysis, innovative teaching, podiatry education

## Abstract

**Background:**

Understanding the structural anatomy and biomechanics of the foot arches is essential for podiatry students due to its clinical relevance in diagnosing conditions such as Pes Planus and Pes Cavus. The foot arches form a flexible base that supports body weight, absorbs shock and functions as a spring lever during activities such as walking and jumping. Therefore, understanding this anatomical knowledge is fundamental for podiatry students for maintaining foot health and supporting clinical assessment and treatment planning. Traditional pedagogical methods often face limitations due to cost, accessibility and emotional or cultural barriers.

**Methods:**

To address these challenges, the footprint practical activity (FPA) was introduced as a hands‐on, cost‐effective and inclusive pedagogical strategy. Students collected their footprints and analysed two‐dimensional footprints to interpret three‐dimensional foot arch structures using Staheli's and Chippaux–Smirak indices. Educational impact was assessed by comparing overall grades between pre‐ (2020) and post‐implementation (2023) cohorts.

**Results:**

Implementation of the FPA resulted in a 12% improvement in academic outcomes. Student feedback highlighted enhanced anatomical comprehension and appreciation of clinical relevance, supporting the effectiveness of the intervention. The diagnostic tools used in the activity, Staheli's index and the Chippaux–Smirak index, showed consistent classification outcomes.

**Conclusion:**

The finding supports the integration of footprint‐based analysis into lower limb anatomy education as a clinically relevant student‐centred approach. The FPA enhances student engagement, supports anatomical learning and facilitates the teaching of foot arch variation, offering a valuable alternative to traditional pedagogical methods in podiatry anatomy education.

## 1 Introduction

In the podiatry profession, effective clinical management of foot and lower limb conditions, including arch disorders, requires precision in both practical skills and anatomical understanding. A solid knowledge of the skeletal framework and soft tissue architecture of the foot, particularly the arches, is essential for accurate diagnosis and targeted intervention. These arches play a vital role in absorbing impact pressure and stabilising the body during gait and posture. Disorders such as flatfoot (Pes Planus) and high arch (Pes Cavus), which alter load distribution and biomechanical function, often lead to musculoskeletal complications, including tendonitis, postural imbalance and joint pain [1, 2].

Footprint‐based analysis, commonly used in clinical settings to identify these conditions [3], presents a unique opportunity to bridge diagnostic relevance with anatomical education. The newly introduced footprint practical activity (FPA) within the Anatomy of the Lower Limb unit at Queensland University of Technology addresses challenges associated with traditional teaching tools. Although cadaveric dissection and 3D anatomical models offer valuable learning, their use is often constrained by financial cost, cultural and emotional sensitivity, ethical considerations and accessibility [4, 5].

The FPA provides a hands on, cost‐effective, clinically relevant and culturally inclusive alternative. It engages students in interpreting footprint patterns to assess foot arch types, requiring synthesis of 3D anatomical knowledge across multiple weeks and body systems. Similar to cadavers, it allows exploration of anatomical variation, but without emotional or cultural barriers [4]. This makes footprint interpretation an ideal pedagogical tool for an active and engaging learning strategy via visual–spatial abilities in podiatry students [6, 7].

The aim of this study was to evaluate the educational impact of the FPA on podiatry students' understanding of foot arch conditions and their academic performance. This was assessed by comparing overall grades between cohorts before and after the implementation of the FPA (2020 vs. 2023). The analysis revealed a 12% improvement in academic outcomes, which could be partially attributed to the enhanced engagement and comprehension facilitated by the activity. Student engagement and feedback were evaluated qualitatively, and the prevalence of Pes Planus and Pes Cavus among participants was analysed using Staheli's method and the Chippaux–Smirak index [8].

## 2 Methods

A student‐centred footprint practical activity (FPA) was conducted during the week 9 practical sessions of the anatomy of the lower limb unit. Students were not expected to demonstrate clinical diagnostic accuracy but rather begin developing foundational skills in observation, pattern recognition and clinical reasoning. As such, formal assessment of individual diagnostic accuracy was not included by design.

The podiatry students collected their own footprints using a standardised method involving talcum powder [9]. To evaluate the impact of the activity on anatomical understanding, students completed short‐answer assessments on foot arch anatomy in Week 7 (pre‐activity) and Week 13 (post‐activity). Student feedback was collected via an online survey after completion of the activity. To determine the prevalence of different foot arch types among the podiatry students, the teaching team analysed the footprint data using two validated methods: Staheli's plantar arch index and the Chippaux–Smirak index.

### 2.1 Participants

The Bachelor of Podiatry at the Queensland University of Technology in Brisbane is a 4‐year degree program. All students participating in this course undertake foundational anatomy in the first semester, followed by advanced anatomy of the lower limb in the second semester of the first year. The participants in this study were first‐year podiatry students who were offered the opportunity to take part voluntarily, with their decision having no impact on their academic performance. The students were enrolled in the 2022–2023 offering of the LSB235 Anatomy of the Lower Limb course (65 contact hours, including lectures and laboratories, from July to mid‐November). The laboratory sessions accommodate approximately 60 students per session.

For footprint data analysis, students with congenital foot deformities, such as clubfoot or other structural anomalies, were excluded from the study. Additionally, individuals with prominent calluses or corns, which could alter their natural footprint and thus skew the results, were also not included. Finally, students who were either absent during the measurement sessions or who chose not to participate were omitted from this study.

### 2.2 Ethics

Ethical clearance for this study was obtained from the Queensland University of Technology's Human Research Ethics Committee (88790), which rigorously reviewed and approved the research proposal. This approval ensured that all research procedures adhered to the stringent ethical guidelines and standards set forth by the institution, safeguarding the rights, welfare and dignity of all participants involved.

### 2.3 Knowledge Assessment

The students' anatomical knowledge of the foot arches was evaluated through two distinct short written assessments, pre‐ and post‐FPA. The assessments (short answer questions) were conducted in week 7 (pre) of the semester and week 13 (post). The written questions were specifically related to the anatomical knowledge of the foot arches, ensuring that students were tested on critical aspects of foot arch anatomy. This dual approach not only reinforced learning through repeated evaluation but also provided a robust measure of the students' grasp of essential foot arch knowledge gained from the FPA. To assess the effect of FPA on overall students' grade, we also compared the final grades of two cohorts (2020 vs. 2023) with and without including the FPA.

### 2.4 Participant Survey

The survey was conducted only in the 2023 cohort. Fifty‐three anatomy students participated in the FPA in week 9 of the semester. In the following week, students were invited to complete a survey on their experience through the Canvas student portal. The survey was generated with Mentimeter software to ensure anonymity. It comprised five questions, including an open‐ended question designed to evaluate the students' perceptions of the FPA's impact on their educational experience.

### 2.5 Measurements of the Foot

The participants' footprints were collected (2022–2023) using talcum powder and hair styling spray, supervised by a single examiner to ensure consistency. A thick layer of talcum powder was evenly distributed on a tray. Participants then stepped into the tray, coating the soles of their feet with the powder. Immediately afterwards, they pressed their feet onto black paper to create clear footprints. A marker was used to trace an outline around each footprint, ensuring precise documentation of the foot's contours.

The plantar arch index (PAI) was then calculated by the students using Staheli's method [10]. First, the medial tangential line was marked by the most medial points on the metatarsal and heel. The midpoint of this line was calculated, and from this point, a perpendicular line was drawn intersecting the footprint. A second perpendicular line was drawn from the most medial point of the heel to the lateral border of the heel. The section crossing the width of the heel region was designated as (a), whereas the width of the central region of the footprint was recorded as (b). The PAI was calculated in centimetres by dividing the width of the heel region (a) by the width of the central foot region (b) (PAI = a/b). The students' measurements were conducted solely to facilitate their understanding of foot arch conditions and were not recorded.

**FIGURE 1:**
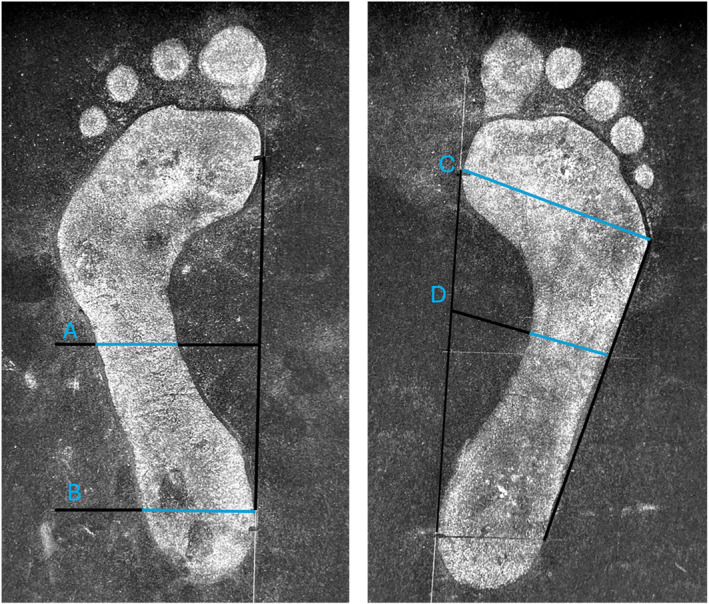
Measurement of the plantar arch index using Staheli's method (ST = a/b) and the Chippaux–Smirak Index (CSI = d/c  ×  100%).

For the prevalence of footprints among podiatry cohort students, an independent researcher (teaching team) employed a similar method to derive the plantar arch index from all collected samples. Using digital images of the footprints and ImageJ 1.54 g [11], the medial tangential line (as above), and lateral tangential line were drawn on the footprints. The lateral tangential line was defined by the most lateral points on the metatarsal and heel (Figure 1). The midpoint of the medial tangential line and the width of the midfoot and heel were ascertained using Staheli's method [10].

The Chippaux–Smirak index (CSI) was calculated as previously explained [12]. The CSI is the ratio between the narrowest width of the midfoot (d) and the widest width of the forefoot (c), calculated as a percentage (CSI = d/c  ×  100%). The metatarsal width (c) was measured from the most lateral point to the most medial point of the forefoot. The width of the midfoot (d) was measured in the middle of the foot, parallel to the metatarsal width (c) (Figure 1).

### 2.6 Statistical Methods

Quantitative data are presented as means, standard deviations and percentages dependent on the nature of the data. Data were analysed via IBM SPSS Statistics (Version 27). A *p‐*value ≤ 0.05 was stated as significant (*p ≤ 0.05). Qualitative data are presented as representative quotes to capture the depth and nuances of the participants' experiences.

## 3 Results

### 3.1 Knowledge Assessment Revealed an Increase Post‐FPA

**FIGURE 2:**
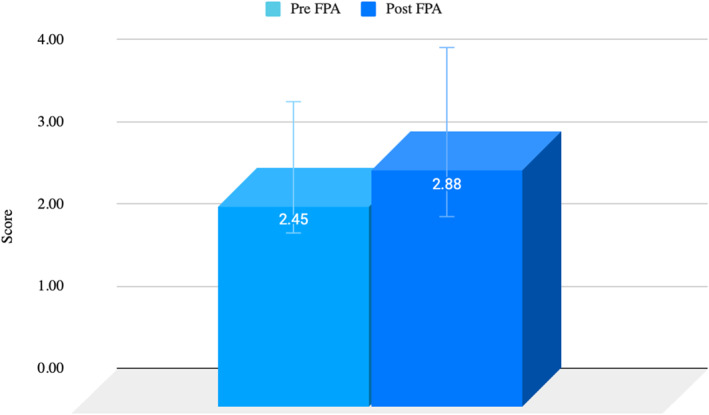
Written knowledge assessment pre‐ and post‐FPA. Post‐FPA knowledge = 2.88 ± 1.03 in comparison to pre‐FPA knowledge = 2.45 ± 0.80; t 53 = 0.02* with a difference between means of 0.43 ± 0.23, 95% CI (0.22, 0.31). Data are expressed as the mean ± standard deviation of the mean (SD); *n* = 53; *p ≤ 0.05; *t*‐test.

We assessed the effects of the footprint practical activity (FPA) on students' written assessment performance regarding anatomical structures and biomechanical knowledge of the foot arches. The FPA was conducted in week 9. Pre‐testing occurred in week 7 with post‐testing in week 13. Objective results based on pre‐ and post‐FPA test results showed an increase in knowledge of 10.72%, with a *t*‐test revealing significant improvement (*p* < 0.05) in total knowledge scores in the domain of foot arch anatomy. Data are outlined in Figure 2.

### 3.2 Improvement of Students' Grades Post‐FPA

**FIGURE 3:**
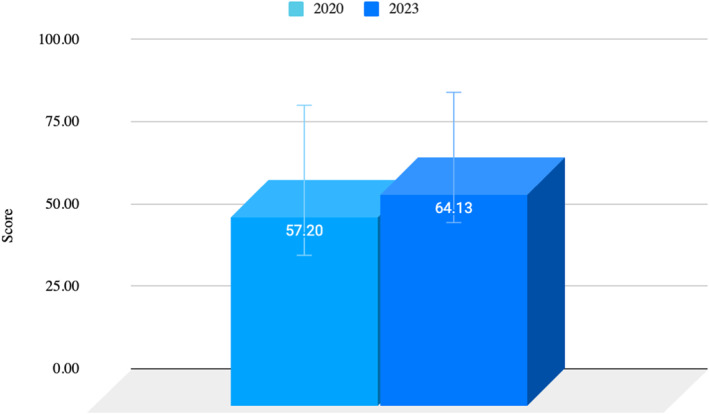
Academic performance: 2020 (before) −2023 (after). 2020 grades = 57.2 ± 22.8 in comparison to 2023 grades = 64.1 ± 19.8; t 91 = 0.13 with a difference between means of −6.93 ± 4.55, 95% CI (−16, 2.12). Data are expressed as the mean ± standard deviation of the mean (SD); *n* = 91; *t*‐test.

We examined student academic performance to evaluate the effects of incorporating the FPA on students' overall grades. Specifically, we compared overall grade averages from the 2020 (before) and 2023 (after) student cohorts (combined *n* = 91), encompassing students aged 18–24 years. Grade data revealed a 12.11% increase in average scores from 2020 to 2023, indicating an upward trend in academic achievement over the studied timeframe. See Figure 3.

### 3.3 Student Survey Highlights Positive Impact of FPA on Learning

**FIGURE 4:**
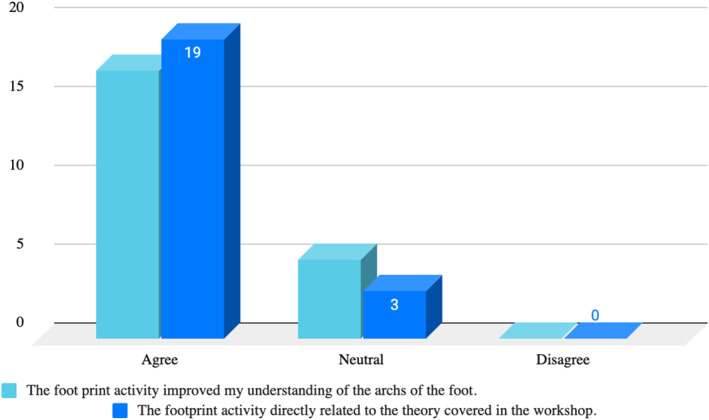
Student survey results on the impact of the FPA. 77% of students agreed that the FPA enhanced their understanding of anatomical structures, whereas 23% were neutral, and 0% disagreed. 86% of students indicated that the FPA content was directly related to the theory covered in the workshop, with 14% remaining neutral and 0% disagreeing.

To evaluate students' responses to the practical experience regarding their engagement and perceived value, a subjective assessment (student survey) was conducted the week following the FPA in 2023. Survey responses were obtained from 35% of the participants (*n* = 21). The majority of students responded positively (e.g., ‘agree’ or ‘strongly agree’) to the questions assessing the impact of the FPA on their comprehension of the arches of the foot (Figure 4). Specifically, 77% of those surveyed agreed that the FPA enhanced their understanding of anatomical structures, whereas 23% of the respondents remained neutral, neither agreeing nor disagreeing with the statement. Notably, none of the students disagreed, suggesting that the activity had a universally positive impact on their learning experience. Additionally, the survey results revealed a strong consensus on the program's relevance, with 86% of students indicating the FPA consolidated content that was covered in the theory, underscoring the program's effectiveness in reinforcing theoretical concepts. Although 14% of the respondents were neutral, neither affirming nor disputing the alignment between the FPA content and the workshop theory, it is noteworthy that no students disagreed.

Students' open‐ended comments were recorded regarding what they most enjoyed about the FPA. The majority of comments reflected a positive student experience. Emerging themes from these comments included an appreciation for the visuospatial aspects of the activity and its student‐centred approach. Comments are listed in Table 1.

**TABLE 1:** Student comments regarding the FPA.

Visuospatial
It visually allowed to me to see the differences between a normal, flat and high arch foot.
Visibly seeing the arch of the foot.
Hands‐on educational exercise to back up theory learnt beforehand seems to work for me.
Student‐centred
I liked that we could utilise our own anatomy and perform an activity that was very practical base.
It was fun to look at my own footprint and calculate the arch index.
Seeing my foot arch.
Measuring the arch.
Fun and practical
Was our first foot assessment of the degree, and while simple, it was relevant and interesting.
The whole experience was really fun.
The practical aspect of the activity. It was helpful in linking theory and practice. Thank you.
Fun to do and was practical.
Helped me learn more and was fun.
Enjoyed doing an activity for a change.
Having a practical based activity to assist with learning and research.
Shows how knowledge can be applied to the body.
It was interactive and easier to retain the information from the topic.

### 3.4 The Rate of Variation of Foot Arch Was Similar Across Measures of ST and CSI

To investigate the prevalence of flatfoot among the podiatry student cohorts, we analysed the footprints from two distinct student cohorts during the years 2022–2023, with a total sample size of 111 participants (*n* = 111). Among these, 69 were female and 42 were male.

The medial longitudinal arch of the foot is commonly used as the basis for classifying arch types, with three primary categories: Pes Cavus, normal arch and Pes Planus. Pes Cavus is characterised by an abnormally elevated arch, resulting in minimal contact between the plantar surface and the ground. This produces a distinct footprint with a narrow midfoot region and a pronounced gap along the medial border. In contrast, a normal arch displays a balanced configuration, neither excessively high nor collapsed. This results in partial contact of the medial midfoot with the ground, typically visualised as a footprint with a moderate concavity along the medial edge. Pes Planus, or flatfoot, is defined by a diminished or absent arch, allowing nearly the entire plantar surface to contact the ground. This is reflected in a footprint with little to no medial concavity and near‐complete foot outline visibility. For comparison, Staheli's index (ST) and Chippaux–Smirak index (CSI) ratios were categorised into three separate groups.

ST classifications were Pes Cavus (< 0.5), normal arch (0.5–0.9) and Pes Planus (> 0.9) [10]. CSI classifications were Pes Caves (< 25%), normal arch (25%–45%) and Pes Planus (> 45%) [12, 13]. The prevalence of flat feet was 18.02% using both ST and CSI methods of measurement, demonstrating comparable validity and reliability between the two methods (see Table 2). Both indices show most participants had normal arched feet, with 73%–76% of participants falling into this category. Pes Cavus was the least common, with 6%–9% of the total population exhibiting this condition. 27.38% of males and 12.32% of females had ST > 0.9, relaying a preponderance of flat feet in the male cohort.

**TABLE 2:** Comparison of foot arch distribution in males and females using the Stahelis arch index and Chippaux–Smirak index.

	*N*	% Pes Cavus	% Normal arch	% Pes Planus
Stahelis Arch Index				
Total	111	8.56	73.42	18.02
Female right foot	69	4.35	84.06	11.59
Female left foot	69	4.35	82.61	13.04
Male right foot	42	4.76	66.67	28.57
Male left foot	42	14.29	59.52	26.19
Chippaux–Smirak Index				
Total	111	6.31	75.68	18.02
Female right foot	69	7.25	81.16	11.59
Female left foot	69	8.70	78.26	13.04
Male right foot	42	4.76	69.05	26.19
Male left foot	42	14.29	57.14	28.57

## 4 Discussion

The footprint practical activity (FPA) was associated with a 12% improvement in podiatry students' academic performance, based on comparative cohort data from 2020 and 2023. Although this suggests that the activity contributed to enhanced understanding of foot arch anatomy and its clinical relevance, causal inference is made with caution due to potential confounding variables such as variations in teaching staff, differences in students' prior knowledge and the ongoing effects of the COVID‐19 pandemic. Future studies with more controlled designs, such as within‐cohort comparisons, may strengthen causal claims.

Survey responses indicated that students found the FPA helpful in consolidating theoretical content and improving spatial reasoning. Although the response rate was limited and therefore the results are indicative rather than definitive, no negative feedback was recorded. Students reported that the activity supported their learning preferences and improved their ability to interpret anatomical structures from footprint data. These findings are consistent with prior research advocating for student‐centred interactive approaches in anatomy education [14].

Limitations of this study include the absence of a contemporaneous control group and the potential for self‐selection bias, as more motivated students may have been overrepresented among survey respondents. It is essential to acknowledge that foot biomechanics are inherently complex, and translating simplified assessments such as footprint analysis using talcum powder into meaningful clinical decision‐making presents significant challenges due to the dynamic and multifactorial nature of foot function [15, 16]. Therefore, the FPA is designed purely as an introductory learning exercise to help first‐year students develop fundamental skills in observation, measurement and data recording.

The FPA offers a cost‐effective alternative to cadaveric dissection and high‐fidelity 3D models, which are often limited by financial, ethical and logistical constraints [4, 5]. Unlike donor tissue, which requires complex preservation and consent protocols, footprint analysis is low‐cost, non‐invasive and accessible to all students. It also avoids cultural and emotional barriers associated with human tissue use [4], making it suitable for diverse educational settings.

Importantly, the activity allows students to observe anatomical variation in real time, reinforcing the concept that structural diversity is common and clinically significant. This mirrors the educational value of donor tissue, where variation is a key learning outcome [17, 18] but without the associated limitations.

Diagnostic analysis using Staheli's index and the Chippaux–Smirak index yielded consistent results, confirming their reliability for screening Pes Planus and Pes Cavus [8, 19, 20]. These methods provided students with direct experience in applying clinical tools, bridging theoretical anatomy with practical diagnostic skills. Future iterations of the FPA could incorporate individual assessments or structured marking rubrics to evaluate diagnostic competence in conjunction with foundational skills.

The FPA represents an early‐stage educational innovation, and this study serves as a foundation for more rigorous evaluation and potential adaptation across broader settings in the future. While the FPA introduces foundational diagnostic techniques, future iterations could incorporate advanced modalities such as imaging to better reflect clinical workflows [21]. This would support a more comprehensive understanding of foot biomechanics and pathology.

Overall, the FPA demonstrates that low‐cost, clinically relevant and inclusive teaching strategies can effectively enhance anatomical education and diagnostic readiness in podiatry students.

## 5 Conclusion

This study suggests that the FPA may serve as a cost‐effective and pedagogically sound tool for enhancing anatomical education in podiatry. This activity appeared to support students' academic outcomes, spatial reasoning and the early development of diagnostic skill development through clinically relevant tasks. By enabling real‐time observation of anatomical variation and eliminating barriers associated with cadaveric materials, the FPA offers an accessible and inclusive practical activity for teaching anatomy of the foot region. Future iterations may benefit from integrating advanced diagnostic modalities to further align with clinical practice and deepen student engagement.

## Author Contributions

**Angela Jacques:** formal analysis, writing – original draft, writing – review and editing. **Fatemeh Chehrehasa:** conceptualization, data curation, writing – review and editing.

## Funding

School of Biomedical Sciences Startup Grant: 241548.

## Ethics Statement

This study was approved by the Queensland University of Technology under approval number 88790.

## Conflicts of Interest

The authors declare no conflicts of interest.

## Data Availability

The data that support the findings of this study are available upon reasonable request from the corresponding author.
